# Research note: approach rather than avoidance: behavioral responses of broiler chicks repeatedly exposed to an autonomous mobile robot

**DOI:** 10.1016/j.psj.2026.107959

**Published:** 2026-10-14

**Authors:** Jorge Martin Caliva, Jackelyn Melissa Kembro, Romina Paula Andrea Picco, Diego Alberto Guzman, Raul Hector Marin

**Affiliations:** a Universidad Nacional de Córdoba (UNC), Facultad de Ciencias Exactas, Físicas y Naturales, Instituto de Ciencia y Tecnología de los Alimentos (ICTA), Córdoba, Argentina; b Consejo Nacional de Investigaciones Científicas y Técnicas (CONICET), Instituto de Investigaciones Biológicas y Tecnológicas (IIByT, CONICET-UNC), Córdoba, Argentina

**Keywords:** Broiler welfare, Mobile robot, Familiarization, Fear response, Environmental enrichment

## Abstract

Previous experience shapes animal behavior, and repeated exposure to non-aversive stimuli can reduce responses to subsequent challenges. Autonomous robots that navigate commercial broiler houses are being developed for environmental monitoring and have been shown to increase bird locomotion during their passage. However, whether this increased activity primarily reflects displacement or fear-induced avoidance, and how robot exposure influences behavioral responses, remains unclear. We assessed behavioral responses of broilers repeatedly exposed to the AviSense robot (Appelie Robotics) compared with non-exposed controls. From day 1 post-hatch, chicks were reared under commercial conditions in three 7.5 × 4.5 m pens containing approximately 500 birds each: one robot-exposed (RE) and two unexposed controls (CON). RE birds were exposed hourly to the robot for 10 min, whereas CON birds remained unexposed to it. At 13-15 d, chicks underwent individual behavioral tests; responses were analyzed with mixed-effects models. In a two-choice runway containing the robot and an unfamiliar basketball at opposite ends, RE chicks left the neutral zone sooner (*P* = 0.013) than controls. Treatment × Zone interaction P-values were 0.060 for latency to enter the stimulus close zones and 0.043 for time spent in these close zones. Fisher’s LSD post hoc comparisons showed that RE chicks entered the robot close zone sooner than CON chicks (P = 0.004), spent more time in that zone than CON chicks (P = 0.004), and spent more time in the robot than the ball close zone (P = 0.036). In tests conducted without the robot, RE chicks showed shorter latency to ambulate in an open field (*P* = 0.002) and required more attempts to induce tonic immobility (*P* = 0.012), whereas social-reinstatement behavior was similar between treatments. Overall, previous robot exposure was associated with a robot-specific increase in subsequent proximity and with lower behavioral inhibition in independent fear-related contexts, providing no evidence that such exposure induced fear-driven avoidance. Repeated exposure to a robot may therefore promote familiarization and facilitate its integration into the birds’ environment.

## Introduction

Autonomous technologies are increasingly being developed for poultry production to support environmental monitoring, flock management, and other labor-intensive tasks. Among these technologies, mobile robots are particularly relevant from an animal-welfare perspective because, unlike stationary sensors, they navigate within the birds' living space and repeatedly interact with the flock. Experimental studies have shown that ground robots can operate among broiler chickens with limited behavioral disruption and can increase bird activity (Dennis et al., 2020; Yang et al., 2020; Marin et al., 2024). Previous studies further suggest that broiler responses to mobile robots can vary with bird age and robot operating conditions (Parajuli et al., 2020; da Rocha Balthazar et al., 2024). Consequently, understanding how broilers respond to and adapt to these devices is important for interpreting the behavioral consequences of their use in commercial poultry houses.

In a previous study conducted under commercial conditions, the passage of the autonomous robot AviSense (Apelie Robotics) rapidly altered broiler spatial distribution and promoted locomotion throughout the growing cycle (Marin et al., 2024). Birds moved away from the robot's zone of influence within seconds, but also interacted physically with the robot and showed ground-directed exploratory behaviors after it passed. Although these observations demonstrate that a mobile robot can stimulate broiler activity, the behavioral mechanism underlying this response remains unknown. Increased locomotion could reflect physical displacement as the robot moves through occupied space or fear-induced avoidance if birds perceive the approaching device as threatening. These possibilities are not necessarily mutually exclusive: immediate displacement or short-range avoidance during robot passage could coexist with experience-dependent changes in how birds subsequently respond to the device. In this regard, repeated non-harmful exposure to the robot as chicks could lead to habituation, with the device becoming a predictable and non-aversive component of the birds’ environment. Reports of shorter avoidance distances with more frequent robot exposure, together with greater escape distances at higher robot speeds (Parajuli et al., 2020; da Rocha Balthazar et al., 2024), support the possibility that both previous experience and robot movement characteristics shape these responses.

Previous experience can also modify chickens' behavioral responses beyond the context in which it occurs. For example, environmental enrichment involving repeated exposure to novel stimuli can reduce behavioral inhibition and avoidance across novel-object, open-field, and tonic-immobility tests (Jones and Waddington, 1992). Thus, repeated exposure to a mobile robot could influence not only the birds' subsequent response toward the device but also their behavioral reactivity in other contexts.

The present study therefore compared broiler chicks exposed to an autonomous mobile robot from early life with non-exposed controls to determine whether previous robot exposure influenced subsequent voluntary approach toward the device and behavioral responses in other contexts. We hypothesized that repeated early robot exposure would promote familiarization rather than persistent fear-related avoidance. If repeated exposure to the robot were predominantly aversive, previously exposed birds would avoid voluntarily approaching the robot and show increased fear-related responses in independent behavioral tests.

## Materials and methods

### Animals, housing, and robot exposure

Female Cobb-500 broiler chicks were obtained from a commercial hatchery and housed under commercial conditions in a 200 × 15 m broiler house. On day 1, chicks were randomly allocated to three 7.5 × 4.5 m pens established within the house, each containing a water and a feeder line and housing approximately 500 chicks per pen (initial stocking density approximately 14.8 chicks/m^2^). Two pens served as unexposed controls (CON), and another pen was assigned to robot exposure (RE). Feed and water were provided ad libitum. The house was equipped with blackout side curtains, fan ventilation, and an evaporative cooling system, as described previously (Marin et al., 2024). Lighting, temperature, and general management followed standard commercial practices. All animal procedures were approved by the Institutional Committee for the Care and Use of Laboratory Animals (CICUAL) of the FCEFyN-UNC (Acta 15).

Beginning on day 1, pens were assigned to either a robot-exposure (RE) or control (CON) treatment. Birds assigned to the RE treatment were repeatedly exposed to an AviSense autonomous mobile robot (Figure 1) (Appelie Robotics S.A.S., Córdoba, Argentina), whereas CON birds were maintained under the same housing and management conditions without robot exposure. The robot was white and measured 38 × 31 × 16.2 cm (L × W × H), weighed approximately 6.2 kg, and had a ground clearance of 4–8 cm. A conspicuous white frontal light, consisting of four LEDs (9,000–10,000 K; 3 W), produced approximately 260–280 lumens over a 120–140° angle. The robot was equipped with a programmable autonomous navigation system using onboard computer vision and, in the present study, moved at approximately 0.05 m/s along a predefined oval trajectory within each pen.

**Fig. 1:**
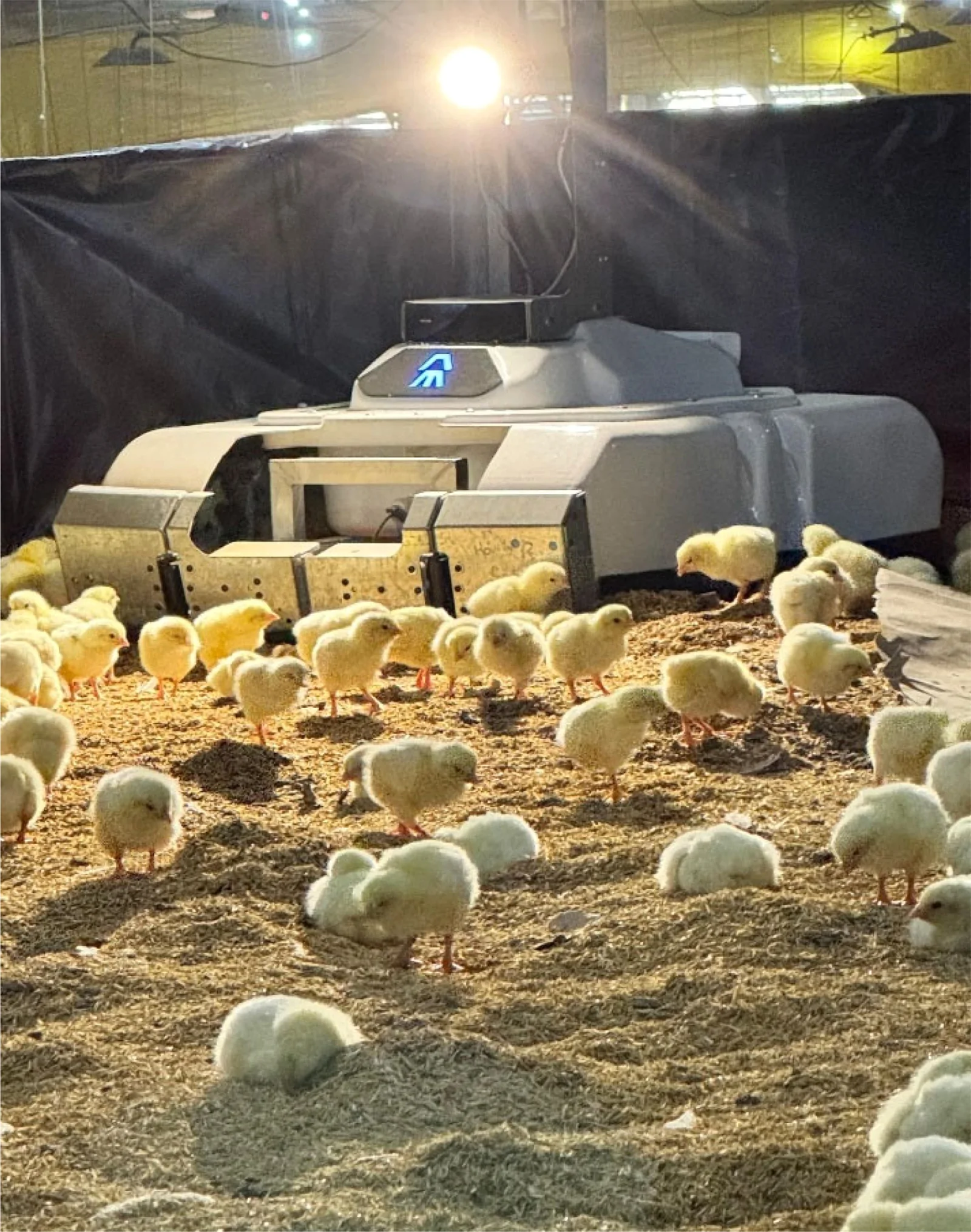
AviSense autonomous mobile robot operating among broiler chicks under commercial rearing conditions.

Robot-exposure sessions were conducted throughout the study, hourly between 09:30 and 16:30 h, with each session lasting approximately 10 min. During each session, the robot autonomously followed the predefined trajectory among the birds without additional human intervention. Control birds were not exposed to the robot during the rearing period.

### Behavioral tests

Behavioral testing was conducted at 13-15 d of age. The two-choice runway test was performed at 13 d, the open-field and tonic-immobility tests at 14–15 d, and the social-reinstatement test at 15 d. Each chick was subjected to only one behavioral test and was subsequently removed from the experimental population to prevent retesting. Mean stocking density across pens progressively decreased from approximately 14.8 to 11.8 chicks/m² by the end of testing at 15 d of age.

*Two-Choice Runway*. Fifty chicks from both treatments were evaluated for their responses toward the robot and an unfamiliar object in a 3-m-long, 32-cm-wide two-choice runway with solid white walls (50 cm high) and the same rice hull litter used in the pens covering the floor. The AviSense robot was positioned at one end of the runway and an orange basketball (24 cm diameter) at the opposite end. The basketball was unfamiliar to all chicks, whereas the robot was familiar to RE chicks (because of their previous exposure) but unfamiliar to CON chicks. No goal boxes or barriers separated the stimuli from the chicks. Thus, a close zone was defined for each stimulus object (robot close zone and ball close zone), extending from 20 cm in front of the object, around the object itself, to the corresponding end of the runway. A plastic wire mesh barrier was used to define a 30-cm wide neutral zone at the center of the runway for a 1-min habituation period. After removal of this barrier, the chicks were allowed to move freely for 3 min. A camera (Ezviz, model: CS-C3TN) was positioned 2.5 m above the runway to record behaviors. Video recordings were subsequently processed using Any-maze software to determine latency to leave the neutral zone, total time spent in the neutral zone, latency to enter the robot and ball close zones, and time spent in each close zone. Four videos from the RE treatment group were lost due to file corruption during download.

*Open-field test.* Thirty-six chicks from both treatments were individually tested in a 100 × 100 × 50 cm open-field arena (Jones, 1977a) with a floor covered with rice hull litter, and solid white wooden walls. Each bird was placed in a corner of the square arena, facing the wall. Its behavior was recorded for 5 min using a camera positioned 2 m above the arena. Using Any-maze software we determined the latency to ambulate, total distance traveled, mean locomotor speed, time spent mobile, and time spent immobile.

*Tonic immobility.* The responses of 126 chicks from both treatments were evaluated in this trial. Two experimenters conducted the tests in parallel. Each chick was placed in a supine position and gently manually restrained for 15 s on a wooden table surrounded by a U-shaped white wall that prevented visual contact with conspecifics (Marin et al., 2001). Following release, tonic immobility (TI) was considered successfully induced when the bird remained immobile for at least 10 s. If TI was not induced, the procedure was repeated, up to a maximum of five induction attempts. The number of attempts required to induce TI and TI duration, measured from release until the bird returned to an upright position, were recorded. The maximum observation time for TI was 180 s. Birds in which TI was not induced after five attempts were excluded from the TI-duration analysis. The proportion of birds requiring five induction attempts and the proportion of birds in which TI could not be induced after five attempts were also determined.

*Social reinstatement test.* Social reinstatement behavior of 90 birds was evaluated individually for 3 min in one of two one-way runways (200 × 30 × 40 cm; L x W x H), made entirely of white wood (Marin et al., 2001). Two stimulus conspecifics were positioned in a 20-cm-long compartment behind a transparent partition at one end of the apparatus. The test chick was positioned in a similar compartment at the opposite end of the runway. After a 1-min habituation period, this partition was removed, allowing the bird to move freely within the apparatus for 3 min. Excluding the 20-cm stimulus compartment, the 180-cm test runway was divided into start (20 cm), distant (70 cm), intermediate (70 cm), and social close (20 cm) zones. Recorded variables were latency to leave the start zone, latency to enter the social close zone, and time spent in the social close, intermediate, and distant zones.

### Statistical analysis

Sample size for each test was estimated from preliminary data considering a power of 80%, and ranged from 20 to 60 depending on the behavioral test. Pen was the experimental unit for robot exposure, with individually tested chicks constituting subsamples within pens. Behavioral responses were analyzed using generalized linear mixed models, with treatment (RE vs. CON) included as a fixed effect and pen as a random effect. Gamma distributions were used for continuous positive behavioral variables when appropriate. For the Two-Choice Runway, time spent in and latency to enter the two stimulus close zones were analyzed using linear mixed-effects models, with treatment (RE vs. CON), zone (robot vs. ball), and their interaction as fixed effects. Bird identity was included as a random effect to account for repeated measurements of the two zones within the same bird. Fisher's LSD tests were used for pairwise comparisons among treatment × zone combinations. Proportional outcomes, including the proportion of birds requiring five TI induction attempts and the proportion in which TI could not be induced, were compared between treatments using tests of equality of proportions. For the latency to enter and time spent in the two-choice runway close zones, *P* ≤ 0.05 was used solely to assign superscript groupings based on Fisher’s LSD comparisons among treatment x zone combinations in Table 1. Exact *P*-values are reported throughout to allow readers to interpret the strength of evidence.

**Table 1:** Behavioral responses of control and robot-exposed broiler chicks.

Behavioral tests	Variables	Control	Robot Exposed	P-values
Two-choice runway test	Latency to leave the neutral zone (s)	61.84±15.32	23.94±11.27	0.013
	Time spent in the neutral zone (s)	85.12±12.59	30.76±7.00	0.001
	Latency to enter the robot close zone (s)	124.08^a^±12.55	63.81^b^±16.57	0.060
	Latency to enter the ball close zone (s)	132.75^a^±13.38	117.55^a^±17.43	
	Time spent in the robot close zone (s)	11.70^b^±3.10	38.56^a^±10.56	0.043
	Time spent in the ball close zone (s)	17.20^b^±6.07	17.50^b^±6.29	
Open-field test	Latency to ambulate (s)	64.42±25.94	21.6 ± 6.75	0.002
	Total distance traveled (m)	4.59±1.00	4.76±1.15	0.95
	Mean speed (cm/s)	4.81±1.18	4.76±1.62	0.98
	Time mobile (s)	88.3 ± 23.58	84.11±16.77	0.95
	Time immobile (s)	212.33±25.94	213.55±15.96	0.85
Tonic immobility	Number of TI induction attempts	2.82±0.16	3.67±0.26	0.012
	TI duration (s)	98.35±7.73	103.00±13.75	0.765
	Proportion of birds requiring 5 induction attempts	0.18	0.52	0.001
	Proportion of birds in which TI was not induced	0.11	0.38	0.001
Social-reinstatement test	Latency to leave start zone (s)	71.72±9.56	87.75±14.34	0.51
	Latency to enter social close zone (s)	96.94±9.36	59.47±11.52	0.28
	Time spent in social close zone (s)	27.25±5.04	23.65±8.86	0.95
	Time spent in intermediate zone (s)	38.68±5.51	33.32±9.52	0.09
	Time spent in distant zone (s)	41.91±6.51	31.66±8.18	0.75

Values are presented as mean ± SE, except proportional outcomes, which are presented as proportions. For latency to enter and time spent in the two-choice runway close zones, tabulated *P*-values refer to the Treatment × Zone interaction. For each of these outcomes, superscript letters indicate Fisher’s LSD post hoc pairwise comparisons among the four treatment x zone combinations across both rows and columns; means lacking a common superscript differ at *P* ≤ 0.05. For all other outcomes, *P*-values refer to CON-RE comparisons.

## Results and discussion

Results of all behavior tests are shown in Table 1. In the Two-Choice Runway test, chicks previously exposed to the robot left the neutral (central) zone sooner (*P* = 0.013) and spent less time in this zone during the test (*P* = 0.001) than CON chicks. Responses toward the two stimulus zones were further evaluated considering treatment (RE vs. CON) and zone (robot vs. ball) simultaneously. Time spent in the close zones varied according to the Treatment × Zone interaction (estimate ± SE = 26.56 ± 13.15 s; z = 2.02, *P* = 0.043). Fisher's LSD comparisons showed that RE chicks spent more time in the robot close zone than in the ball close zone (*P* = 0.036) and more time in the robot close zone than CON chicks (P = 0.004). In contrast, time spent in the ball close zone was similar between RE and CON chicks (*P* = 0.974; Table 1). The Treatment × Zone interaction for latency to enter the stimulus close zones yielded an estimate ± SE of −45.07 ± 23.95 s (z = −1.88, *P* = 0.060). Fisher's LSD comparisons showed that RE chicks reached the robot close zone sooner than the ball close zone (*P* = 0.003) and had shorter latencies to the robot close zone than CON chicks (*P* = 0.004). In contrast, latency to reach the ball close zone was similar between RE and CON chicks (*P* = 0.469; Table 1). Together, these responses suggest that previous robot exposure specifically altered the chicks' subsequent response toward the robot rather than producing generalized changes toward either stimulus. The shorter latency to reach the robot and the greater time spent near it are consistent with experience-dependent familiarization and reduced avoidance. Habituation to repeated robot exposure could contribute to reduced avoidance. The voluntary approach toward the stationary robot in this test is also consistent with the device having become a familiar stimulus, rather than merely with a reduced response to its presence. This interpretation may be particularly relevant because the test was conducted in a novel environment, where the familiar robot could have provided a recognizable stimulus that facilitated approach. Familiar cues have previously been shown to reduce behavioral inhibition in chicks exposed to novel environments (Jones, 1977b), while avoidance responses toward mobile robots have been reported to decrease with repeated exposure (Parajuli et al., 2020). However, an additional contribution of exploratory attraction cannot be excluded. During repeated exposure in the home pens, the moving robot and its frontal light provided dynamic visual and physical stimulation that may have promoted exploration and interaction with the device. Notably, during the Two-Choice Runway test, the robot was stationary and its light was switched off, suggesting that the enhanced approach observed in the RE chicks cannot be attributed to the immediate effects of these features during testing itself; instead, it most likely reflects previous experience with the robot as a familiar and behaviorally salient stimulus.

The responses observed in behavioral tests conducted in the absence of the robot provide complementary information. In the open-field test, robot-exposed chicks showed a shorter latency to first movement than controls (*P* = 0.002; Table 1), whereas total distance traveled, mean speed, time mobile, and time immobile did not differ between treatments (*P* ≥ 0.85). The shorter latency to initiate movement therefore does not appear to reflect a generalized increase in locomotor activity but rather lower fear-related behavioral inhibition when birds were introduced into the novel open-field environment. A complementary pattern was observed in the tonic-immobility test. Robot-exposed chicks required more induction attempts than controls (*P* = 0.012), and greater proportions required the maximum of five induction attempts or could not be induced into TI (*P* = 0.001 for both). Among chicks in which TI was successfully induced, TI duration did not differ between treatments (*P* = 0.765). Together, the greater difficulty in inducing TI after manual restraint and the shorter latency to move in the open field shown by the RE chicks are consistent with reduced expressions of fear-related responses in these test situations (Jones and Faure, 1981; Jones and Waddington, 1992). However, shorter latency to ambulate and greater difficulty in inducing TI do not necessarily mean that RE chicks were less fearful in other situations.

Similar effects have been reported following environmental enrichment, which increased activity in an open field and attenuated tonic-immobility responses in domestic chicks (Jones and Waddington, 1992). This parallel raises the possibility that repeated robot exposure provided an enrichment-like stimulation. The robot repeatedly introduced movement and visual stimulation into an otherwise relatively predictable and plain rearing environment. As noted in the introduction, previous observations under commercial conditions (Marin et al., 2024) also showed that its passage not only displaced broilers locally but was also associated with physical interactions with the device and subsequent ground-directed exploratory behaviors. These responses suggest that robot passage may elicit behavioral activity extending beyond the immediate locomotor response to the approaching device. Although the present experiment was not designed to establish whether an autonomous robot constitutes environmental enrichment, repeated interaction with such a dynamic and salient stimulus could certainly provide additional sensory and behavioral stimulation. Importantly, this interpretation does not exclude local displacement or short-range avoidance during robot passage. Birds may move away from the robot as it approaches while, with repeated non-harmful exposure, becoming familiar with the device and more willing to approach it voluntarily.

No detectable treatment differences were observed in the social-reinstatement test. Latency to leave the start zone, latency to enter the social close zone, and time spent in the social close, intermediate, and distant zones did not differ between treatments (all *P* ≥ 0.09; Table 1). Hence, this test provided no evidence of repeated robot exposure altering social-reinstatement behavior or the motivation to regain proximity to conspecifics. Together with the other behavioral tests, these findings suggest that differences associated with previous robot exposure were not expressed uniformly across behavioral contexts. Further studies quantifying individual differences in interactions with robots and evaluating both sexes across ages and rearing conditions would help assess the broader applicability of these findings.

Overall, the observed responses are consistent with familiarization with the robot and do not support persistent fear-driven avoidance in the behavioral tests evaluated. The shorter latency to approach the robot and greater time spent near it after repeated exposure, together with differences in fear-related responses across the distinct contexts of a novel environment and physical restraint, are consistent with repeated robot exposure being behaviorally well tolerated and suggest that it may have provided enrichment-like sensory and exploratory stimulation. However, because robot exposure was evaluated in a pen, potential pen-specific contributions to the observed differences cannot be excluded. These initial findings suggest that, beyond their monitoring and management functions, autonomous mobile robots could offer opportunities for behavioral stimulation compatible with broiler welfare.

## Ethics declaration

### Animal subject

This study was conducted in accordance with the following guidelines for animal welfare and/or reporting: Guide for the Care and Use of Laboratory Animals: Eighth Edition. (Washington, DC: The National Academies Press). This study was approved by the Institutional Committee for the Care and Use of Laboratory Animals (CICUAL) of the FCEFyN-UNC (Acta # 15).

## Declaration of generative AI and AI-assisted technologies in the writing process

Declaration of Generative AI and AI-Assisted Technologies in the Writing Process: During the preparation of this work, the authors used ChatGPT (OpenAI) to assist with language editing and to improve grammar, clarity, and readability. After using this tool, the authors reviewed and edited the content as needed and take full responsibility for the content of the publication.

## Author contributions

All authors participated in the conception of the research, development of the study design and protocol, and planning of the overall research methodology. JMC, JMK, DAG, and RHM conducted the behavioral trials. JMC and RPAP performed the statistical analyses. JMC, JMK, and RHM prepared the initial manuscript draft. JMK and RHM secured funding supporting the study. All authors critically reviewed the manuscript for important intellectual content, read and explicitly approved the final version, and agree to be personally accountable for their contributions and for ensuring that questions related to the accuracy or integrity of any part of the work are appropriately investigated and resolved.

## Disclosures

The authors declare that they have no known competing financial interests or personal relationships that could have appeared to influence the work reported in this paper.
